# Racial/ethnic and prior willingness disparities in potential living kidney donors’ self-assessed responses to *advancing American kidney health* regulation

**DOI:** 10.1186/s12889-021-12023-w

**Published:** 2021-11-01

**Authors:** Selena E. Ortiz, Ashton M. Verdery, Jonathan Daw

**Affiliations:** 1 Health Policy and Administration, Demography, and Public Policy, Department of Health Policy and Administration, The Pennsylvania State University, 604N Donald H. Ford Building, University Park, PA 16801 USA; 2 Sociology and Demography, Department of Sociology and Criminology, The Pennsylvania State University, University Park, USA

**Keywords:** Health policy, Health disparities, Kidney transplantation, Racial/ethnic minorities

## Abstract

**Background:**

Racial/ethnic disparities in living donor kidney transplantation (LDKT) are large, and rates of LDKT may be limited by indirect costs of living donation. A 2019 Executive Order– Advancing American Kidney Health (AAKH)– sought to remove indirect costs through an expanded reimbursement program. We examine how potential living kidney donors in the U.S. believe regulation stemming from the AAKH initiative will impact their living donor evaluation likelihood, how these beliefs vary by minority race/ethnicity and prior willingness to be evaluated, and how differences are explained by ability to benefit or knowledge and attitudes.

**Methods:**

Data from a 2019 online survey (Families of Renal Patients Survey) were used. Respondents are U.S. adult (> 18 years) members of the Qualtrics Survey Panel who reported having relatives with weak or failing kidneys (*N* = 590). Respondents’ likelihood to be evaluated for living kidney donation are measured by self-report. Prior willingness is measured by past donation-related actions and current attitudes. Ability to benefit is measured by self-reported labor force participation and financial strain. Transplant knowledge is measured by self-report and a knowledge test, and transplant-related attitudes are measured by self-report. Average marginal effects of minority race/ethnicity and prior willingness for response to each provision in fully-adjusted models were estimated. Formal tests of mediation were conducted using the Karlson, Holm, and Breen (KHB) mediation model. Stata/MP 14.2 was used to conduct all analyses.

**Results:**

Majorities of all groups report favorable responses to the provisions stipulated in AAKH regulation. Responses to provisions are significantly associated with race/ethnicity and prior willingness, with racial/ethnic minorities and those not previously willing to be evaluated less likely to report favorable responses to these provisions. Prior willingness differences are partially explained by group differences in ability to benefit and transplant-related knowledge and attitudes, but racial/ethnic differences largely are not.

**Conclusions:**

Regulation stemming from the AAKH initiative is likely to effectively promote LDKT, but may also exacerbate racial/ethnic disparities. Therefore, the regulation may need to be supplemented by efforts to address non-financial obstacles to LDKT in racial/ethnic minority communities in order to ensure equitable increases in LDKT rates and living donor support.

**Supplementary Information:**

The online version contains supplementary material available at 10.1186/s12889-021-12023-w.

## Background

End-stage kidney disease (ESKD) is a significant burden on U.S. population health and health policy systems, as its prevalence reached 2242 per million in 2018 and accounted for 7.2% of Medicare’s budget [1]. Living donor kidney transplantation (LDKT) is often the optimal therapy for ESKD patients, as it is associated with significantly better prognosis than deceased donor kidney transplants (DDKTs) or dialysis [1, 2]. Despite these benefits, LDKTs are much rarer than DDKTs, and actually declined as a share of kidney transplants from 2004 to 2017 [3]. Racial/ethnic disparities in LDKTs are significant—in 2019, Black patients represented 31.5% of the kidney transplant waiting list but only 13.1% of LDKT recipients, while White patients represented 37.9% of the transplant waiting list but 63.9% of LDKT recipients [2].

LDKTs are shaped not only by patients’ own medical and social circumstances, but those in their social network as well. Since they represent a majority of living kidney donors (61.3% in 2014), transplant candidates’ family members are a crucial population of interest for living kidney donation [4]. Key factors in family members’ likelihood to donate may include their own health, blood and tissue compatibility with the patient, LDKT-related knowledge and attitudes, and trust in medical institutions [5–9].

Furthermore, while donation surgery and follow-up care are covered by the patient’s insurance, donors frequently face substantial financial consequences. In 2019, the *Advancing American Kidney Health* (AAKH) Executive Order (E.O.) sought to remove the various indirect costs incurred by living kidney donors [10]. For example, donors who are geographically distant from transplant clinics must cover travel costs associated with medical evaluations and surgery; donors who are employed but are without paid medical leave must forgo wages during convalescence; and donors with caretaking responsibilities must secure and cover the costs of child and elder care during recovery. These costs can be substantial even with financial assistance, with a third of respondents to a recent study reporting net costs exceeding $2500 [11]. These costs may deter many from donating a kidney, and disproportionately deter racial/ethnic minorities. One study found that the Black-White disparity in living kidney donation is restricted to the lowest neighborhood income quintile; for the top three neighborhood income quintiles, Black living kidney donation rates are higher than those for Whites [12]. These findings suggest that financial barriers are a major driver of racial/ethnic disparities in LDKT. The AAKH mitigates these barriers via regulation that broadens an existing program to reimburse living kidney donors for their travel-related expenses, increase the income limit for expense reimbursement eligibility, and expand the definition of reimbursable LDKT expenses to include kidney-donation-related lost wages, child-care, and elder-care expenses [13, 14].

The efficacy of regulation stemming from the AAKH E.O. and its impact on LDKT racial/ethnic disparities will hinge on which members of the potential donor population are most impacted by it — if only the most likely donors are affected, the regulations’ impact may be minimized; if the impact varies across racial/ethnic groups, LDKT disparities could increase, even if all groups benefit compared to the status quo. We aimed to assess whether the expanded LDKT financial neutrality provisions stipulated in the regulation (hereinafter ‘provisions’) are associated with self-assessed change in likelihood to be evaluated for living kidney donation (hereinafter ‘response to the provisions’), how response to the provisions varies across categories of race/ethnicity and prior willingness to be evaluated for living kidney donation, and whether these categorical differences in response to the provisions are explained by ability to benefit from these provisions. Since non-financial obstacles may also impact categorical differences in LDKT, we also test whether differences are explained by transplant-related knowledge and attitudes such as trust in medicine and religious objections to transplantation.

## Methods

### Data

We examine data from the Families of Renal Patients Survey (FoRPS) Wave 2, an online, non-probability, 57-item survey. The FoRPS emerged from a prior survey examining living kidney donation processes and determinants from the perspective of both transplant candidates and their family members. Survey items come from established government surveys such as the American Community Survey or Current Population survey, or are previously-validated survey items related to kidney transplantation. All other survey items were developed by the authors. The survey was administered between August 27 and September 1, 2019.

U.S. adults age 18 and older were recruited through the Qualtrics Survey Panel. Respondent eligibility included 1) ever having a family member diagnosed with “weak or failing kidneys” and 2) having an eligible relationship with that individual (see the Supplementary Digital Content for information about Qualtrics Survey Panel recruitment and full details on FoRPS respondent eligibility, survey instrument, and additional analyses).

The study was reviewed and approved by the institutional review board of The Pennsylvania State University.

### Measures

#### Response to the AAKH provisions

Respondents were asked to read a brief description of the AAKH E.O.: *“A recently signed Executive Order, “Advancing American Kidney Health,” directs the Secretary of Health and Human Services to propose regulation to remove financial barriers associated with living organ donation, including costs stemming from the evaluation, hospitalization, surgery, follow-up care, and treatment of any surgical complications for living organ donors.”* Respondents were then asked to rate how each of the following provisions would change their likelihood of being evaluated for living kidney donation: Travel and Food Reimbursement, Raised Income Limit for reimbursement, Dependent Care Reimbursement related to donation, and donation-related Lost Wage Reimbursement. Respondents rated their likelihood to be evaluated for living kidney donation on a 5-point scale from “much more likely to be evaluated” to “much less likely to be evaluated”, which we recoded into three categories (“more likely to be evaluated,” “neither more nor less likely to be evaluated,” or “less likely to be evaluated”).

#### Race/ethnicity

Respondent race/ethnicity was measured by self-report. To measure respondent racial/ethnic identity, respondents were asked, *“Which of the following describes your racial and ethnic identity? Check all that apply,”* with the following options: “Caucasian or White”, “Hispanic or Latino/a”, “Black or African-American”, “Asian or Pacific Islander,” “American Indian or Alaska Native,” or “Other.” Due to our moderate sample size, individual racial/ethnic minority groups were too small to analyze separately (*N* = 97 Black, 53 Hispanic, 30 Asians/Pacific Islander, and 27 other respondents). In the Supplementary Digital Content, we show that detailed racial/ethnic identity among racial/ethnic minorities is not statistically significantly associated with our primary outcome, so our race/ethnicity analyses distinguish between Whites and all other groups (hereinafter “minority race/ethnicity”).

#### Prior willingness

We measured prior willingness to be evaluated for living kidney donation (hereinafter “prior willingness”) by whether respondents had agreed to be medically evaluated, completed medical evaluation, been approved, and/or donated their kidney (coded “Concrete Yes”). Among respondents not reporting any of these events, those who indicated that they would be evaluated for living kidney donation if asked were coded “Hypothetical Yes,” and those who did not were coded “No.” These measures were collected prior to the regulation response measures in the FoRPS, and are therefore unaffected by the AAKH information.

#### Ability to benefit

The provisions address financial constraints on LDKT behaviors that may not apply to all potential donors. Because lost wage reimbursement is more beneficial to those who work and work more hours, labor force participation status in the prior week was measured in four categories: working full-time, working part-time, those without paid work, and those with a job but not at work last week. Because financial reimbursement may be more important for those under greater financial strain, financial strain was measured using a cluster of measures related to respondents’ ability to afford a variety of expenses — housing, childcare, eldercare, vehicle, healthcare, education, and other expenses — if they or a family member had to take a month off work. Each item was measured on a 3-point scale: “not at all difficult”, “somewhat difficult”, and “very difficult”. These items were summed together in two separate indices: an index of dependent care financial strain (the sum of childcare and eldercare strain items) and other expense financial strain (the sum of other strain items).

#### Transplant-related knowledge and attitudes

FoRPS measured self-rated transplant knowledge as well as a battery of items measuring respondents’ objective transplant-related knowledge [6, 8]. Self-rated knowledge about issues related to transplantation was measured on a 5-point scale, from “none at all” to “a great deal.” Objective transplant-related knowledge was measured by five factual transplant-related questions with multiple choice response sets, with each item including a “Don’t know” response option. The number of correct responses was summed together, treating “Don’t know” as an incorrect answer. The survey also included measures of respondents’ trust in medical institutions (=1 if “a great deal” of trust and = 0 if otherwise) and whether they have religious objections to transplantation (=1 if agree their religious beliefs make them uncomfortable with transplantation and = 0 if otherwise).

#### Controls

All regression analyses were adjusted for self-reported respondent gender, age, and political identification. For the gender item, respondents could mark “Male”, “Female”, or “Other”; due to limited “Other” responses (*n* = 13), this category was combined with “Male.” Analyses reported in the Supplementary Digital Content show that combining this group with “Female” did not statistically significantly change the results. Respondent age is operationalized in four categories: 29 or younger, 30–49, 50–69, and 70 or older. Because our dependent variables are responses to regulatory provisions, we control for political identification in three categories: Democrat/lean Democrat, Republican/lean Republican, and Independent/other.

### Statistical analysis

**Fig. 1:**
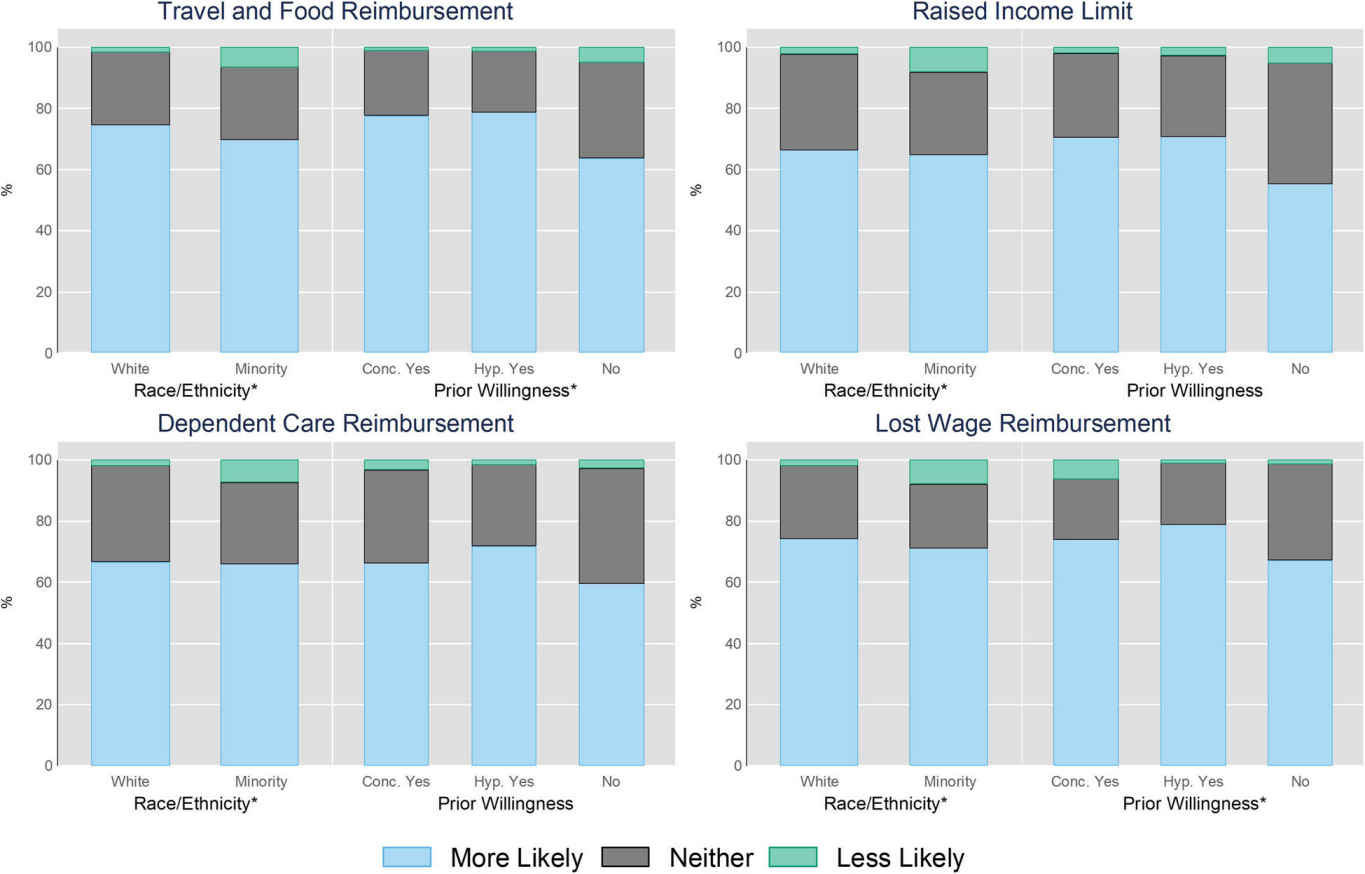
Likelihood of being Evaluated for Living Kidney Donation by Provision, Race/Ethnicity, and Prior Willingness to Donate^1,2^. ^1^Indicates respondents’ likelihood of being evaluated for living kidney donation per each provision. ^2^Indicates whether respondents had agreed to be medically evaluated, completed medical evaluation, been approved, and/or donated their kidney (‘Concrete Yes’); whether respondents would be evaluated for living kidney donation if asked (‘Hypothetical Yes’); or whether respondents would not be evaluated (‘No’). Note: Asterisks indicate that the results of a weighted χ2 test were statistically significant (*p* < .05) (see the Supplemental Digital Content for more information)

Post-stratification weights by patient race/ethnicity, gender, and age group are used to make the weighted sample comparable to the U.S. ESKD patient population [15]. We present the analysis in three stages. First, we describe the weighted sociodemographic characteristics (Table 1) and the weighted transplant-related attitudes and characteristics of the overall sample (Table 2). Second, we describe the weighted distribution of likelihood to be evaluated for living kidney donation, presented separately by provision, respondent race/ethnicity, and prior willingness (Fig. 1). We assess the statistical significance of group differences using weighted χ^2^ tests.

**Table 1:** Respondent Sociodemographic Characteristics, Families of Renal Patients Study Wave 2

Variable	N	Weighted Prop./Mean
Race/Ethnicity		
White	382	0.488
Minority	208	0.512
Sex		
Male	229	0.363
Female	361	0.637
Respondent’s Age		
18–29	153	0.251
30–49	288	0.48
50–69	136	0.25
70+	13	0.02
Respondent’s Political ID		
Democrat	239	0.477
Republican	192	0.266
Independent	159	0.257
Labor Force		
Employed Full-Time	298	0.509
Employed Part-Time	82	0.128
Unemployed or OOLF	178	0.303
Other	32	0.059
Patient’s Relation to Respondent		
Parent	163	0.307
Significant Other	122	0.153
Grandparent	88	0.17
Sibling	46	0.079
Aunt/Uncle	86	0.182
Child	15	0.007
Cousin	34	0.049
Other	36	0.053

‘OOLF’ stands for out of the labor force

**Table 2:** Respondent Transplant-Related Attitudes and Characteristics, Families of Renal Patients Study Wave 2 (*N* = 590)

Variable	Variable Range	Weighted Prop./Mean
Financial Strain		
Dependent Care Expenses	2–6	3.695
Other Expenses	5–15	9.611
Transplant Knowledge		
Self-Rated Knowledge	1–5	2.803
Knowledge Test Score	0–5	1.699
Attitudes		
Medical Trust	0–1	0.455
Religious Objections	0–1	0.183
**Variable**	**Category N**	**Weighted Prop./Mean**
Provisions^a^		
*Travel & Food*		
Less Likely	24	0.053
Neither more nor less likely	136	0.233
More Likely	430	0.714
*Raise Income Limit*		
Less Likely	34	0.067
Neither more nor less Likely	170	0.281
More Likely	386	0.652
*Dependent Care*		
Less Likely	27	0.069
Neither more nor less Likely	168	0.288
More Likely	395	0.643
*Lost Wages*		
Less Likely	25	0.046
Neither more nor less Likely	136	0.237
More Likely	429	0.717
Prior Willingness^b^		
Concrete Yes	210	0.318
Hypothetical Yes	207	0.354
No	173	0.328

^a^Indicates respondents’ change in likelihood of being evaluated for living kidney donation per each provision. All provisions variables are coded in three categories, and the ‘Category N’ column reflects each cell’s subsample size

^b^Indicates whether respondents had agreed to be medically evaluated, completed medical evaluation, been approved, and/or donated their kidney (‘Concrete Yes’); whether respondents would be evaluated for living kidney donation if asked (‘Hypothetical Yes’); or whether respondents would not be evaluated (‘No’). This variable is coded in three categories and the ‘Category N’ column reflects each cell’s subsample size

**Fig. 2:**
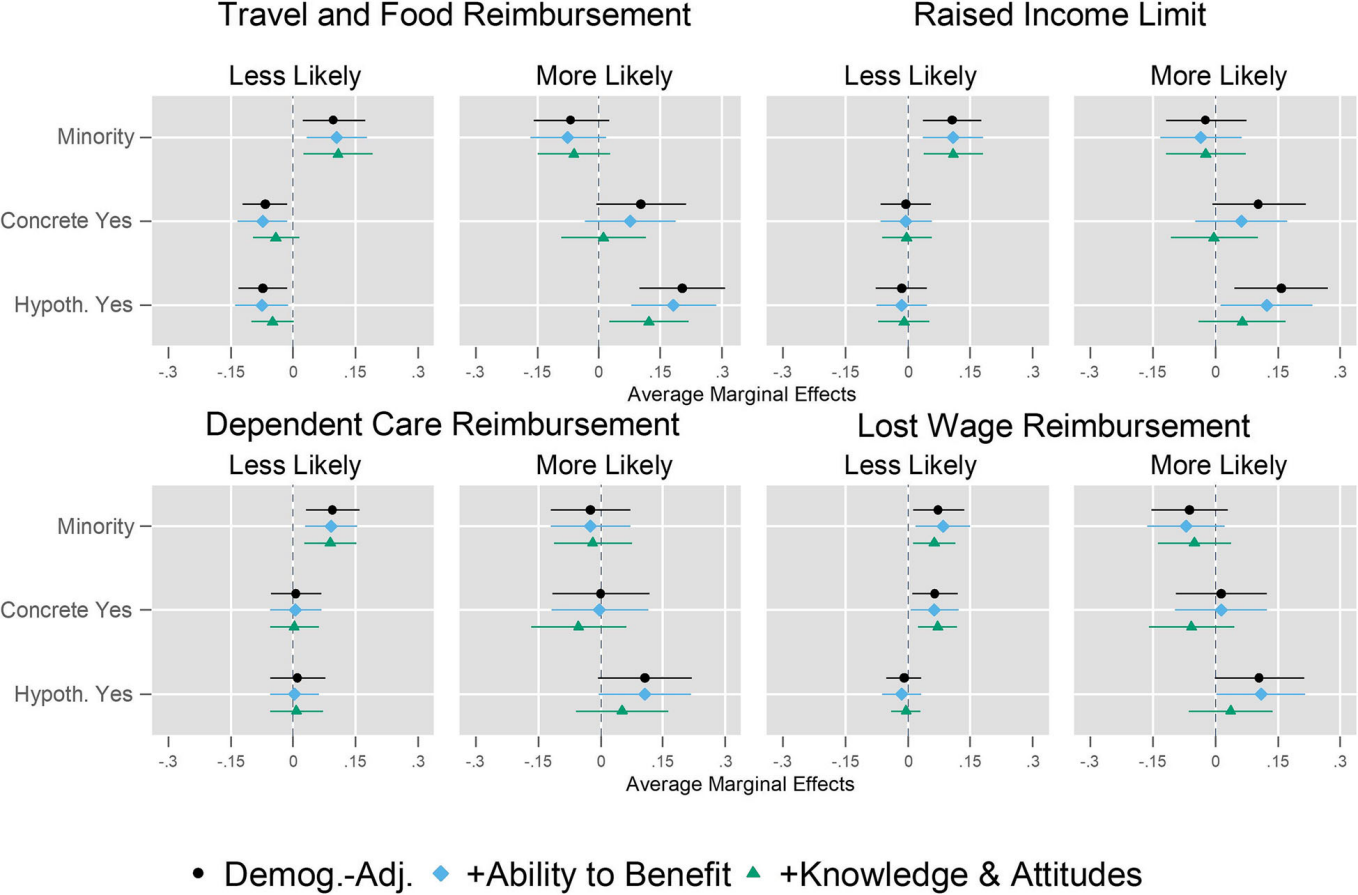
Average Marginal Effects of Minority Race/Ethnicity and Prior Willingness to Donate on “Less Likely to be Evaluated” and “More Likely to be Evaluated” by Provision^1,2^. ^1^Indicates respondents’ likelihood of being evaluated for living kidney donation per each provision. ^2^Indicates whether respondents had agreed to be medically evaluated, completed medical evaluation, been approved, and/or donated their kidney (‘Concrete Yes’); whether respondents would be evaluated for living kidney donation if asked (‘Hypoth. Yes’); or whether respondents would not be evaluated (‘No’). Note: The average marginal effects are calculated based on weighted χ^2^ tests performed to test the relationship of each dependent variable with race/ethnicity and prior willingness (see the Supplemental Digital Content for more information). ‘Demog.-Adj.’ stands for sociodemographic controls, including age, gender, and political identification. Ability to benefit is measured by labor force status, dependent care expense strain, and other expense strain. Knowledge is self-assessed and measured through a factual test. Attitudes include trust in medicine and religious objections to transplantation

Third, we assess how well ability to benefit and transplant-related knowledge and attitudes explain racial/ethnic and prior willingness differences in response to the provisions, net of age, sex, and political identification. The results of this analysis are presented in two ways. In Fig. 2, we present three estimated average marginal effects (AMEs) of minority race/ethnicity and prior willingness on each regulation response, from three different model specifications: with sociodemographic and political adjustments only, with added controls for ability to benefit, and with added controls for transplant-related knowledge and attitudes. In Table 3, we report formal mediation tests using the Karlson, Holm, and Breen (KHB) mediation model [16–19], presenting AMEs from models with and without the mediating variables in each row, their *p*-values, and the estimated percentage of the baseline effect explained.

**Table 3:** Contributions of Demographic Characteristics, Ability to Benefit, and Knowledge and Attitudes to Racial/Ethnic and Prior Willingness Differences in Regulation Responses

Mediation Model^**a**^	Ind. Var.	Less Likely					More Likely				
		Red. Coef.	P	Full Coef.	P	% Med.	Red. Coef.	P	Full Coef.	P	% Med.
**Travel and Food Reimbursement**											
Bivariate + Demog.-Adj.	Minority^b^	0.063	***0.002***	0.067	***0.002***	−5.9	− 0.028	0.453	− 0.033	0.409	−17.9
	Conc. Yes^c^	− 0.032	0.096	− 0.036	0.067	−13.2	0.098	***0.018***	0.097	***0.022***	1.1
	Hyp. Yes^c^	− 0.053	***0.026***	− 0.055	***0.023***	−3.9	0.168	***0.000***	0.168	***0.000***	− 0.1
Demog.-Adj. + Ability to Benefit	Minority	0.059	0.961	0.082	***0.001***	−38.7	−0.027	0.494	−0.039	0.325	−44.0
	Conc. Yes	−0.027	0.995	−0.042	0.066	−54.6	0.101	***0.016***	0.080	0.064	21.4
	Hyp. Yes	−0.049	0.987	−0.063	***0.022***	−29.1	0.172	***0.000***	0.153	***0.001***	11.2
Demog.-Adj. + Knowledge & Attitudes	Minority	0.064	***0.003***	0.069	***0.002***	−7.1	−0.026	0.498	− 0.030	0.437	−14.9
	Conc. Yes	−0.038	0.054	−0.024	0.232	37.6	0.094	***0.022***	0.030	0.482	67.7
	Hyp. Yes	−0.057	***0.017***	−0.046	0.052	19.3	0.168	***0.000***	0.104	***0.016***	37.9
Demog.-Adj. + Ability to Benefit + Knowledge & Attitudes	Minority	0.058	0.936	0.082	***0.001***	−42.2	−0.023	0.544	− 0.031	0.419	−33.8
	Conc. Yes	−0.029	0.982	−0.030	0.195	−5.4	0.094	***0.021***	0.027	0.539	71.9
	Hyp. Yes	−0.048	0.955	−0.052	0.050	−7.8	0.168	***0.000***	0.099	***0.022***	40.8
**Raised Income Limit**											
Bivariate + Demog.-Adj.	Minority	0.078	***0.000***	0.074	***0.001***	4.3	−0.013	0.750	−0.030	0.493	− 130.0
	Conc. Yes	−0.008	0.726	−0.011	0.621	−43.1	0.080	0.082	0.071	0.133	11.8
	Hyp. Yes	−0.019	0.425	−0.020	0.396	−7.5	0.145	***0.002***	0.141	***0.003***	2.5
Demog.-Adj. + Ability to Benefit	Minority	0.073	***0.001***	0.078	***0.001***	−5.8	−0.023	0.599	−0.040	0.351	−77.7
	Conc. Yes	−0.013	0.583	−0.008	0.737	38.5	0.076	0.105	0.043	0.360	42.6
	Hyp. Yes	−0.022	0.362	−0.018	0.455	18.3	0.146	***0.002***	0.119	***0.013***	18.8
Demog.-Adj. + Knowledge & Attitudes	Minority	0.077	***0.001***	0.074	***0.001***	3.6	−0.027	0.524	−0.031	0.466	−14.8
	Conc. Yes	−0.011	0.628	−0.013	0.593	−16.4	0.068	0.134	−0.002	0.962	103.3
	Hyp. Yes	−0.020	0.397	−0.018	0.457	9.3	0.141	***0.002***	0.069	0.145	51.1
Demog.-Adj. + Ability to Benefit + Knowledge & Attitudes	Minority	0.076	***0.001***	0.077	***0.001***	−1.5	−0.024	0.568	− 0.037	0.377	−55.2
	Conc. Yes	−0.012	0.597	−0.011	0.644	7.7	0.070	0.121	−0.011	0.811	116.3
	Hyp. Yes	−0.021	0.378	−0.017	0.497	20.4	0.142	***0.002***	0.060	0.208	58.0
**Dependent Care Reimbursement**											
Bivariate + Demog.-Adj.	Minority	0.095	0.967	0.070	***0.002***	26.2	0.006	0.876	−0.001	0.985	113.3
	Conc. Yes	0.020	0.518	0.013	0.558	35.6	0.031	0.499	0.028	0.557	11.5
	Hyp. Yes	−0.003	0.997	−0.013	0.592	− 380.6	0.111	***0.020***	0.108	***0.025***	2.4
Demog.-Adj. + Ability to Benefit	Minority	0.071	***0.002***	0.069	***0.002***	2.1	0.003	0.942	−0.003	0.939	205.8
	Conc. Yes	0.014	0.507	0.014	0.525	2.3	0.030	0.530	0.019	0.689	35.4
	Hyp. Yes	−0.013	0.593	−0.013	0.605	1.9	0.110	***0.022***	0.101	***0.039***	8.3
Demog.-Adj. + Knowledge & Attitudes	Minority	0.071	***0.002***	0.067	***0.003***	5.3	0.000	0.991	0.003	0.938	824.3
	Conc. Yes	0.013	0.555	0.009	0.688	29.1	0.024	0.599	−0.023	0.629	195.8
	Hyp. Yes	− 0.014	0.565	− 0.014	0.580	1.7	0.108	***0.022***	0.054	0.263	49.3
Demog.-Adj. + Ability to Benefit + Knowledge & Attitudes	Minority	0.072	***0.001***	0.067	***0.003***	6.3	0.002	0.970	0.004	0.933	− 120.3
	Conc. Yes	0.014	0.526	0.010	0.646	24.1	0.025	0.596	−0.023	0.639	192.7
	Hyp. Yes	−0.015	0.547	−0.014	0.578	4.6	0.107	***0.023***	0.054	0.273	49.7
**Lost Wage Reimbursement**											
Bivariate + Demog.-Adj.	Minority	0.055	***0.003***	0.063	***0.002***	−13.7	−0.032	0.390	−0.035	0.376	−10.2
	Conc. Yes	0.063	***0.016***	0.066	***0.013***	−4.5	0.029	0.508	0.036	0.410	−27.1
	Hyp. Yes	0.002	0.944	0.004	0.884	− 106.6	0.094	***0.035***	0.099	***0.028***	−5.3
Demog.-Adj. + Ability to Benefit	Minority	0.066	0.411	0.072	***0.001***	−8.2	−0.032	0.415	−0.040	0.316	−23.3
	Conc. Yes	0.099	0.968	0.068	***0.014***	31.8	0.039	0.379	0.032	0.478	18.2
	Hyp. Yes	0.040	0.988	0.002	0.951	95.2	0.102	***0.024***	0.097	***0.034***	4.7
Demog.-Adj. + Knowledge & Attitudes	Minority	0.064	***0.002***	0.060	***0.003***	6.2	−0.034	0.378	−0.023	0.555	33.0
	Conc. Yes	0.066	***0.012***	0.064	***0.016***	3.1	0.031	0.465	−0.013	0.776	140.5
	Hyp. Yes	0.005	0.878	0.005	0.874	−4.3	0.096	***0.027***	0.044	0.333	54.8
Demog.-Adj. + Ability to Benefit + Knowledge & Attitudes	Minority	0.066	0.644	0.068	***0.002***	−2.1	−0.033	0.398	−0.024	0.534	26.2
	Conc. Yes	0.102	0.990	0.064	***0.021***	37.0	0.032	0.457	−0.009	0.836	129.1
	Hyp. Yes	0.043	0.996	0.001	0.966	96.9	0.096	***0.027***	0.047	0.297	51.1

^a^All analyses were conducted using the Karlson, Holm, and Breen (KHB) method

^b^Reference category is ‘White’

^c^Reference category is ‘No’

*P*-values statistically significant at the *p* < 0.05 level are highlighted in bold and italics. ‘Ind. Var.’ stands for independent variable; ‘Conc. Yes’ stands for concrete yes; ‘Hyp. Yes’ stands for hypothetical yes; ‘Red. Coef.’ stands for reduced model coefficient; ‘Full Coef.’ stands for full model coefficient; ‘% Med.’ stands for percent mediated. ‘Minority’ represents all racial/ethnic minorities in the sample, including Black, Hispanic, Asian/Pacific Islander, American Indian or Alaska Native, and Other. ‘Demog.-Adj.’ stands for sociodemographic controls, including age, gender, and political identification. Ability to benefit from AAKH regulatory provisions is measured by labor force status, dependent care expense strain, and other expense strain. Knowledge is self-assessed and measured through a factual test. Attitudes include trust in medicine and religious objections to transplantation

Stata/MP 14.2 (Stata Corp, College Station, TX) was used to conduct all analyses. An alpha level of 0.05 was used for all 2-tailed statistical tests. All analyses were conducted in winter 2021 and were carried out in accordance with relevant guidelines and regulations.

## Results

A total of 590 U.S. adults who have a relative diagnosed with weak or failing kidneys completed the survey (response rate 62.1%). Fifty-one percent of our weighted sample were racial/ethnic minorities. The gender distribution skewed female (64%). Forty-eight percent of the sample were aged 30–49, with only 2% aged > 70. Forty-eight percent of the sample identified as Democrat and 51% had full-time employment. Thirty-one percent of the sample reported thinking about a parent with weak or failing kidneys.

### Transplant-related attitudes and characteristics

Respondents reported moderate to high levels of dependent care (3.7 mean/6 max) and other (9.6 mean/15 max) financial strain. Respondents rated their transplant-related knowledge as moderate (2.8 average/5 max), and on average answered about one-third of the factual questions correctly. Forty-six percent of respondents had “a great deal” of trust in medical institutions, while 18% agreed that their religious views made them less likely to donate a kidney.

A majority of respondents reported “more likely to be evaluated” in response to all provisions (71% for Travel and Food Reimbursement, 65% for Raised Income Limit, 64% for Dependent Care Reimbursement, and 72% for Lost Wage Reimbursement). High proportions of respondents indicated some level of prior willingness to be evaluated for living kidney donation—32% “Concrete Yes” and 35% “Hypothetical Yes”.

### Racial/ethnic minority and prior willingness differences in response to AAKH provisions

Overall, responses were equally distributed across racial/ethnic and prior willingness categories. Positive responses are common for all provisions in all subcategories – at least 60% of every racial/ethnic and prior willingness subcategory reported “more likely to be evaluated”, and only small percentages reported “less likely to be evaluated” for each provision.

However, racial/ethnic minorities had statistically significantly less favorable response to the provisions than Whites. These differences are substantively moderate and vary across provisions and the direction of change. For instance, racial/ethnic minorities have a lower probability of reporting “more likely to be evaluated” in response to Travel and Food Reimbursement and a higher probability of reporting “less likely to be evaluated” than Whites (*p* = 0.001). The Raised Income Limit provision elicits “more likely to be evaluated” responses at roughly equal rates in both racial/ethnic categories, but racial/ethnic minorities have a higher probability of reporting “less likely to be evaluated” than Whites (*p* = 0.001). Similar patterns are observed by race/ethnicity for Dependent Care Reimbursement (*p* < 0.001) and Lost Wage Reimbursement (*p* = 0.022).

Prior willingness categories also differed in response. Across all provisions, a majority of the “No” prior willingness group reported “more likely to be evaluated”, but even higher proportions in the “Concrete Yes” and “Hypothetical Yes” groups reported “more likely to be evaluated”. These differences were statistically significant for Travel and Food Reimbursement (*p* = 0.003) and Lost Wage Reimbursement (*p* = 0.034). The “Hypothetical Yes” group reported consistently more favorable response to both Travel and Food Reimbursement and Lost Wage Reimbursement than the “No” group. The “Concrete Yes” group responded more favorably than the “No” group to Travel and Food Reimbursement but less favorably than the “No” group in response to Lost Wage Reimbursement.

### Ability to benefit and transplant-related knowledge and attitudes

We demonstrate whether group differences in ability to benefit or transplant-related knowledge and attitudes explain racial/ethnic and prior willingness differences. Figure 2 graphically assesses this possibility while Table 3 decomposes the contribution of these factors to these categorical differences in response to the provisions. Broadly, these results show that ability to benefit and transplant-related knowledge and attitudes moderately contribute to prior willingness differences in response to the provisions, but contribute little to racial/ethnic differences.

Ability to benefit partially explains the higher probability that “Concrete Yes” and “Hypothetical Yes” groups report “more likely to be evaluated” in response to Travel and Food Reimbursement. Similar but stronger patterns are found for “more likely to be evaluated” responses to the Raised Income Limit, Dependent Care Reimbursement, and Lost Wage Reimbursement provisions.

However, adjusting for ability to benefit does little to alter the racial/ethnic difference in likelihood of being evaluated. For 6 of the 8 outcomes studied, controlling for ability to benefit actually increases residual racial/ethnic differences in response to the provisions. The two exceptions are both for Dependent Care Reimbursement, where the differences in probability are trivial.

Adjusting for transplant-related knowledge and attitudes presents a similar pattern, as it partially explains prior willingness differences in response to the provisions, but does little to explain racial/ethnic differences. For two provisions (Raised Income Limit, Lost Wage Reimbursement), the demographically-adjusted “Concrete Yes” AME for “more likely to be evaluated” is reduced to zero or reversed once transplant-related knowledge and attitudes are accounted for. In the case of “more likely to be evaluated” responses to Travel and Food Reimbursement, this is sufficient to eliminate the previously statistically significant effect of the “Concrete Yes” group. For “less likely to be evaluated” responses, the contribution of transplant-related knowledge and attitudes to the “Concrete Yes” effect is generally moderate for Travel and Food Reimbursement and Dependent Care Reimbursement; but negligible for the Raised Income Limit and Lost Wage Reimbursement provisions. Similarly, the contributions of transplant-related knowledge and attitudes to the “Hypothetical Yes” group’s responses are uniformly larger for “more likely to be evaluated” responses than “less likely to be evaluated” responses, but smaller in absolute magnitude than the corresponding “Concrete Yes” mediation figures. For three provisions (Raised Income Limit, Dependent Care Reimbursement, and Lost Wage Reimbursement), adjusting for transplant-related knowledge and attitudes renders a previously-statistically-significant association statistically insignificant.

As with ability to benefit, adjusting for transplant-related knowledge and attitudes does little to explain the association between minority race/ethnicity and response to the provisions. The only exception is that this factor mediates the minority race/ethnicity association with “more likely to be evaluated” in response to Lost Wage Reimbursement by 33%. Similar results are obtained when the mediation of both the ability to benefit and transplant-related knowledge and attitudes are assessed simultaneously.

## Discussion

Using a unique dataset of relatives of persons with weak or failing kidneys, we examined the potential for regulation related to the 2019 *Advancing American Kidney Health* E.O. to affect LDKT rates and racial/ethnic LDKT disparities. Maximizing population health and health equity are sometimes conflicting goals in the medium term, as those best positioned to immediately benefit from new medical innovations, resources, or knowledge often already have better health than the general population [20–22]. Our findings show that this may be the case with the provisions related to the AAKH, on the basis of three key findings. First, respondents report that these provisions would increase their likelihood to be evaluated for living donation at very high rates. Second, the majority of respondents who were previously reluctant to be evaluated for kidney donation report that these provisions would increase their likelihood to do so. Together, these first two findings suggest that the provisions are likely to be effective in increasing LDKT rates. Third, however, although the majority of all racial/ethnic groups say that each provision would increase their likelihood to be evaluated for living donation, racial/ethnic minority respondents were statistically and substantively significantly less likely to say so. Furthermore, this disparate response was largely not attributable to their ability to benefit from these provisions or their transplant-related knowledge and attitudes, suggesting that making LDKTs financially neutral or educating potential donors about their benefits are unlikely to solve the issue. This third finding raises the possibility that these provisions may not equally promote all groups’ LDKT rates. Combined with the first two key results, the provisions may therefore result in increased LDKT rates for all racial/ethnic groups *and* increased racial/ethnic LDKT disparities.

### Policy and practice implications

Our findings suggest that racial/ethnic minorities are less likely than Whites to be receptive to the AAKH’s efforts to make LDKT financially neutral. As a result, supplemental efforts may be needed to ensure that all racial/ethnic groups benefit equally from these provisions. To avoid exacerbating disparities, health care providers should continue to work with ESKD patients and families to assess non-financial obstacles to LDKT, particularly among racial/ethnic minority groups. Future research should also continue to address the determinants of donor-side LDKT behaviors beyond financial disincentives and transplant-related knowledge and attitudes. The recommendations of a 2015 consensus conference on disparities in LDKTs offers a useful guide [23]. It recommends financial disincentives to living kidney donation be removed, which the AAKH accomplishes, but also that the transplant community should take concerted steps to further develop culturally-tailored, community-based educational efforts; and establish transplant liaisons between dialysis clinics and transplant centers. As noted in the consensus conference recommendations, however, these interventions have not been widely adopted [23]. These efforts, and the continued efforts of the research community to identify and ameliorate racial/ethnic barriers to LDKT, should continue apace to complement the financial neutrality provisions of the AAKH.

We also note that we ask respondents to self-assess how the AAKH provisions would affect their evaluation behaviors. This does not account for regulation awareness or bureaucratic barriers to reimbursement that donors may encounter. Health care providers and policymakers should ensure that information about these new provisions are broadly distributed, and that the administrative burden required of donors to claim and collect reimbursements are minimized.

### Limitations

This analysis is limited in several respects. First, our study relies on self-report, which may be subject to significant response bias, specifically, social desirability bias. However, this is unlikely to explain the racial/ethnic or prior willingness differences in response to the provisions. Second, our inclusion criteria for the study restricted the sample to family members of kidney disease patients, omitting non-family contacts which are an increasing share of all donors in recent years [2]. This was done purposively—because typical individuals have many more non-family social contacts than family members, we imposed this restriction on sample eligibility to avoid a disproportionate predominance of patients’ friends in the sample. The tradeoff of this decision is that the generalizability of our findings is limited to family members. Future research should therefore examine how these provisions may impact donations from non-family social contacts compared to family members.

## Conclusion

Effectively, ethically, and equitably increasing LDKTs are important goals to promote the health of the ESKD patient population. Our findings indicate that regulation stemming from the *Advancing American Kidney Health* E.O. may achieve the first two goals, but may not achieve the third. As a result, the regulation may need to be supplemented by efforts to address non-financial obstacles to LDKT in racial/ethnic minority communities in order to ensure equitable increases in LDKT rates and living donor support.

## Supplementary Information

**Additional file 1.** Supplementary Digital Content.

## Data Availability

The datasets generated during and analysed during the current study are not publicly available due to limitations of ethical approval involving the patient data but are available from the corresponding author on reasonable request.

## Abbreviations

LDKT: Living donor kidney transplantation
AAKH: Advancing American Kidney Health
ESKD: End-stage kidney disease
DDKT: Deceased donor kidney transplant
FoRPS: Families of Renal Patients Survey
AME: Average marginal effect
KHB: Karlson, Holm, and Breen
