# Supplementary material for: Racial/ethnic and prior willingness disparities in potential living kidney donors’ self-assessed responses to advancing American kidney health regulation
Source: BMC Public Health. 2021 Nov 1;21:1971. doi: 10.1186/s12889-021-12023-w (PMC8561865; doi:10.1186/s12889-021-12023-w)
Supplement: Supplementary file 1 — Additional file 1. Supplementary Digital Content. [file 12889_2021_12023_MOESM1_ESM.docx]

**Supplementary Digital Content**

**Post-Stratification Weights Construction**

We created post-stratification weights that weight the sample to the 2016 ReNDER database. Specifically, we constructed counts of the number of kidney disease patients (i.e., not the respondents, but the relatives they were reporting on) in the analytic sample that fell into groups cross-classified by sex (respondent reported being female vs. respondent reported being male or other), age (less than 20, 20-29, 30-39, 40-49, 50-59, 60-69, 70-79, 80-89, or 90 or older), and race and ethnicity (Hispanic and any other race, non-Hispanic Caucasian or White alone, non-Hispanic Black or African-American alone, non-Hispanic Asian or Pacific Islander alone, or non-Hispanic American Indian or Alaska Native alone or non-Hispanic Other alone or non-Hispanic multiple races). We calculated the same cross-classified counts at the population level based on data in the RenDER system, based on data we obtained on May 10, 2019 on the prevalent population, with all disease modalities, dropping unknown categories, with all diagnoses, and in the U.S. only. Then, we post-stratified the sample data to the population data using Stata’s *svyset* command.

**Survey Content**

Overview

The Families of Renal Patients Survey (FoRPS) Wave 2 is an online, non-probability survey administered to U.S. adults age 18 and older. The FoRPS emerged from a prior survey examining living kidney donation processes and determinants from the perspective of both transplant candidates and their family members. Survey items come from established government surveys such as the American Community Survey or Current Population survey, or are previously-validated survey items related to transplantation. All other survey items were developed by the authors. The FoRPS was fully launched into the field between August 27, 2019 and September 1, 2019.

Participants for FoRPS were recruited through the Qualtrics Survey Panel. Qualtrics is an online survey platform that partners with numerous other survey panels and uses several methodologies to recruit panelists. Participants are recruited from various sources, including website intercept recruitment, member referrals, targeted email lists, gaming sites, customer loyalty web portals, permission-based networks, and social media, etc. Consumer panel members’ names, addresses, and dates of birth are typically validated via third-party verification measures prior to their joining a panel.^[[1]](#footnote-1)^ Qualtrics panelists are compensated through independent partners (e.g. partner-specific rewards, gift cards, etc.), not by the investigators. Because Qualtrics panelists join from a variety of sources, they may receive different types of compensation. They may be airline customers who chose to join the Qualtrics panel in reward for SkyMiles, retail customers who opted in to get points at their favorite retail outlet, or general consumers who participate for cash or gift cards. When panelists are invited by Qualtrics to take a survey, they are informed what they will be compensated by Qualtrics.^1^

Eligibility

U.S. adult members of Qualtrics Survey Panel were invited to participate in the survey. Per Qualtrics’ survey invitation process: “Respondents are invited to surveys in various ways. Often, potential respondents are sent an email invitation informing them that the survey is for research purposes only, how long the survey is expected to take, and what incentives are available. Other times, respondents will see surveys they are likely to qualify for upon signing into a panel portal. Other common invitation methods include in-app notifications and SMS notifications. To avoid self-selection bias, survey invitations do not include specific details about the contents of the survey and are instead kept very general.” ^[[2]](#footnote-2)^

Potential respondents initiated the FoRPS by answering a screener question: *“Before we begin this survey we need to complete a quick check to ensure your eligibility to participate. Think about the living adults in your extended family, including your spouse or partner, children, parents, siblings, grandparents, grandchildren, aunts and uncles, nieces and nephews, cousins,* *and other family members to whom you feel close. To the best of your knowledge, have any of them ever been diagnosed with weak or failing kidneys? Please focus on adult members of your family, and do not include kidney stones, bladder infections or incontinence.”* Those who responded affirmatively to this item were then asked to specify their relationship to this person from the following options: *“My spouse or a boyfriend/girlfriend or life partner who lives with me”; “My child”; “My parent”; “My sibling”; “My grandparent”; “My aunt/uncle”; “My niece/nephew”; “My cousin”; “My grandchild”; “A different type of family member not already indicated (specify).”* Respondents also had the opportunity to select “A different type of family member not already indicated (specify).” Open-ended responses to the “specify” portion of this question were examined to determine respondent eligibility. Responses that could be recoded into one of the categories above were included. We excluded respondents who gave answers that were some variant of “me” or “myself” or otherwise not eligible (e.g., “Godfather,” “Family friend,” “Coworker”).” Of the 971 persons who responded to the initial Qualtrics invitation, 603 completed the survey and passed Qualtrics’s quality controls, and 590 passed both the screener question and indicated a valid, eligible relationship.

All eligible respondents were then asked a series of close-ended questions pertaining to the recipient, including social and demographic characteristics of the recipient; knowledge of

recipient’s kidney disease treatment; and whether respondent and recipient had ever discussed living kidney donation. Respondents were then asked about their own health status, knowledge pertaining to LDKT outcomes, and social and demographic characteristics. Then, the survey ascertained respondents’ level of concern about covering necessary expenses in the event of time off from work and their level of support for policies targeted to ease financial burden for living donors.

Participation Rates

Table S1 summarizes respondents’ participation in the FoRPS survey. 971 members of the Qualtrics Online panel were included in the initial sample because they indicated that they had a relative with weak or failing kidneys. Out of these 971 respondents, 106 (10.9%) were ineligible based on their relationship with the patient (N=865). Out of these 865 eligible respondents, 262 (30.3%) only partially completed the survey, while 603 (69.7%) completed it. Of those that remained, 13 additional cases were eliminated from the analytical sample, for a final sample size of 590. According to the American Association for Public Opinion Research, the most conservative response rate (Response Rate 1) is defined as the number of completed cases (603) divided by the number of contacted individuals (971), for a response rate of 62.1%. Including partial survey responses in the numerator (Response Rate 2) increases the response rate to 89.1%.

Response to the AAKH Provisions

Near the end of the survey, respondents were asked four questions about LDKT-relevant provisions of the Advancing American Kidney Health (AAKH) Executive Order (E.O.). This section began with the following description: *“The following questions refer to your opinions about proposed changes to Federal policies.* *A recently signed Executive Order, ‘Advancing American Kidney Health,’ directs the Secretary of Health and Human Services to propose regulation to remove financial barriers associated with living organ donation, including costs stemming from the evaluation, hospitalization, surgery, follow-up care, and treatment of any surgical complications for living organ donors. Below are some new federal policies that may be included in the proposed regulation. If enacted and you were considering becoming a living kidney donor, please indicate whether each of these regulations would make you much more likely, somewhat more likely, neither more nor less likely, somewhat less likely, or much less likely to be evaluated as a living kidney donor.”*

Respondents were asked about the following provisions: “Reimbursement by the federal government for all food, lodging, and transportation costs associated with living organ donation” (“Travel & Food”), “Raise the limit on the income of donors eligible for reimbursement of food, lodging, and transportation costs associated with living organ donation” (“Raise Income Limit”), “Reimbursement by the federal government for all child and elder care costs associated with living organ donation” (“Dependent Care”), and “Reimbursement by the federal government for lost wages (up to $8000) associated with living organ donation” (“Lost Wages”). The respondents were then asked to rate their self-assessed response to each of the four items on the following scale: “Much more likely”, “Somewhat more likely”, “Neither more nor less likely”, “Somewhat less likely”, “Much less likely”. For the analysis, “Much more likely” and “Somewhat more likely” were combined into “More likely”, and “Much less likely” and “Somewhat less likely” were combined into “Less likely.”

Race/Ethnicity

To measure respondent racial/ethnic identity, respondents were asked, *“Which of the following describes your racial and ethnic identity? Check all that apply,”* with the following options: “Caucasian or White”, “Hispanic or Latino/a”, “Black or African-American”, “Asian or Pacific Islander,” “American Indian or Alaska Native,” or “Other.” To determine whether the number of response categories could be appropriately reduced, we conducted a chi-squared test on the cross-tabulation of race/ethnicity and each regulation response among those with racial/ethnic identities other than non-Hispanic White. Table S2 summarizes these findings. For the four regulation responses examined, none of these tests yielded statistically significant results, suggesting that recoding all racial/ethnic minority groups together into a single category is consistent with our data.

Prior Willingness to Donate

This characteristic of respondents was measured in two stages. First, they were asked, “Continuing to think of your relative with weak or failing kidneys, check all that apply: This person is seeking a kidney transplant; We have discussed me becoming a living kidney donor to this person; I have agreed to be medically evaluated as a potential living kidney donor to this person; I was medically evaluated as a potential living kidney donor to this person; I was medically approved as a potential living kidney donor to this person; I donated my kidney to this person.” If the respondent reported that they had agreed to be evaluated, had been evaluated, had been approved, or had donated their kidney to this person, they were characterized as ‘Concrete Yes’.

If they did not indicate that any of these events had occurred, respondents were asked the following item: “Would you agree to be medically evaluated for donating a kidney to the person you are thinking of if they asked you to?” Respondents who marked “Yes” were characterized as ‘Hypothetical Yes’; respondents who marked no or “don’t know” were characterized as ‘No.’

Transplant-Related Objective Knowledge Details

Based on previously published work,^12,13^ we asked respondents to answer a series of factual questions related to kidney transplantation. We list them here for the reader’s reference, with correct answers marked with an asterisk.

Instructions: The following are a series of questions concerning your knowledge of transplant issues. Please answer them to the best of your ability.

(1) On average, patients who receive a kidney transplant from a living donor:

1. Wait longer for the transplant compared to patients who receive a kidney from someone who has died
2. Wait as long for the transplant compared to patients who receive a kidney from someone who has died
3. Wait less time for the transplant compared to patients who receive a kidney from someone who has died*
4. Don't know

(2) On average, patients who receive a kidney transplant:

1. Live longer than patients who remain on dialysis*
2. Live as long as patients on dialysis
3. Live less long than patients on dialysis
4. Don’t know

(3) On average, a kidney transplanted from a living donor will:

1. Last longer than a kidney from a donor who has died*
2. Last the same amount of time as a kidney from a donor who has died
3. Last less time than a kidney from a donor who has died
4. Don’t know

(4) After the surgery for kidney donation, a living kidney donor will be likely to:

1. Return home about 5 days after surgery*
2. Return home several weeks after surgery
3. Be transferred to a physical rehabilitation center about 5 days after surgery
4. Be transferred to a physical rehabilitation center several weeks after surgery
5. Don’t know

(5) A person over 60 years old cannot be a living kidney donor.

1. True
2. False*
3. Don't know

Sick Relative Demographic Characteristics

The demographic characteristics of the relative with weak or failing kidneys were used to construct post-stratification weights as described above. Each of these items were asked following a survey section header which read, *“The following items refer to characteristics of the relative or family member with weak or failing kidneys that you are thinking about for the survey. You might not know the answer to every question, but please make your best guess from the available options.”* To measure recipient age, respondents were asked, “What is their age in years?” with responses ranging from “Less than 20” to “90 or older,” with 10-year blocks in between. To measure recipient gender, respondents were asked, *“What is their gender?”* with the same response options as above. To measure recipient race/ethnicity, respondents were asked, *“Which of the following describes their racial and ethnic identity? Check all that apply,”* with the same options listed as described above for respondents. However, due to a coding error, respondents were only allowed to mark on racial/ethnic identity for their recipient. To measure recipient educational attainment, respondents were asked, *“What is the highest degree or level of school they have completed?”* and were presented the same response options as above.

**Supplementary Results**

Descriptive Statistics

Table S3 presents the weighted proportions of respondents who reported that each regulatory provision would make them less likely, neither less likely nor more likely, or more likely to be evaluated as a living kidney donor. In our sample, a very high proportion of respondents reported that the lost wages and travel and food provisions would make them more likely to be evaluated as living kidney donors – more than 71% in both cases. Lower, but still quite high, proportions of respondents reported that raising the income limit on reimbursement eligibility (0.652) and reimbursement of child and elder care (0.643) would make them more likely to be evaluated as living donors as well.

Table S4 provides other key weighted descriptive statistics on the sample. Approximately one-third of respondents were coded in each of the willingness to be evaluated categories. 70% of the weighted sample had a high school education or less. Sixty-four percent of the sample were female. Approximately half of the same were racial/ethnic minorities. Turning to the kidney disease patient’s relationship to the respondent, 31% of patients were the respondent’s parent, 18% of patients were the respondent’s aunt or uncle, 17% of patients were the respondent’s grandparent, 15% of patients were the respondent’s significant other, with the remainder of relationships distributed among siblings (8%), cousins (5%), children (1%), and other relationships (5%).

Table S5 provides the detailed statistics underlying Figures 1 and 2 in the main body of this article, and are provided for the reader’s reference. For comparison, results on the full sample and stratified by educational attainment are also provided. The non-parenthetical figures are weighted proportions of the indicated subsample reporting the response in question; the parenthetical figures are the corresponding standard errors.

Hypothesis Test Details

Table S6 provides detailed information on the chi-squared tests performed to test the relationship of each dependent variable with race/ethnicity, education, and prior willingness. Race/ethnicity is statistically significantly associated with all four dependent variables. Education is not statistically significantly associated with any of the four dependent variables, which is why it is omitted from the main analysis. Prior willingness to donate is statistically significantly associated with responses to the Travel and Food and Lost Wage items.

**Supplementary Results Tables**

Table S1: Response Rates, Families of Renal Patients Survey

| Subsample | N | % of Prior Row N |
| --- | --- | --- |
| Total Cases | 971 | -- |
| Eligible | 865 | 89.1% |
| Completed Survey | 603 | 69.7% |
| Retained for Analysis | 590 | 97.8% |

Table S2: Chi-Squared Test Results of Racial/Ethnic Minority Subgroups’ Associations with Regulation Responses

| **Dependent Variable** | **χ^2^** | **P** |
| --- | --- | --- |
| Travel and Food Reimbursement | 2.026 | 0.917 |
| Raised Income Limit | 3.476 | 0.747 |
| Dependent Care Reimbursement | 3.570 | 0.735 |
| Lost Wage Reimbursement | 8.194 | 0.224 |

Note: χ^2^ statistics and p-values were obtained from a cross-tabulation of each regulation response with detailed race/ethnicity among racial/ethnic minorities in FoRPS.

Table S3: Wald Tests Comparing Focal Coefficients with Two Alternative Recodings of Respondent Gender

| **Dependent Var.** | **Regulation Response** | **Independent Var.** | **F** | **P** |
| --- | --- | --- | --- | --- |
| Travel and Food Reimbursement | Less Likely | Minority Race | 0.037 | 0.847 |
|  |  | Conc. Yes | 0.171 | 0.679 |
|  |  | Hyp. Yes | 0.917 | 0.338 |
|  | More Likely | Minority Race | 0.027 | 0.869 |
|  |  | Conc. Yes | 1.328 | 0.249 |
|  |  | Hyp. Yes | 1.097 | 0.295 |
| Raised Income Limit | Less Likely | Minority Race | 0.413 | 0.521 |
|  |  | Conc. Yes | 0.880 | 0.348 |
|  |  | Hyp. Yes | 0.504 | 0.478 |
|  | More Likely | Minority Race | 0.060 | 0.807 |
|  |  | Conc. Yes | 0.480 | 0.489 |
|  |  | Hyp. Yes | 0.273 | 0.601 |
| Dependent Care Reimbursement | Less Likely | Minority Race | 0.347 | 0.556 |
|  |  | Conc. Yes | 0.001 | 0.970 |
|  |  | Hyp. Yes | 0.561 | 0.454 |
|  | More Likely | Minority Race | 0.08 | 0.777 |
|  |  | Conc. Yes | 1.362 | 0.243 |
|  |  | Hyp. Yes | 1.023 | 0.312 |
| Lost Wage Reimbursement | Less Likely | Minority Race | 0.018 | 0.894 |
|  |  | Conc. Yes | 0.704 | 0.402 |
|  |  | Hyp. Yes | 0.462 | 0.497 |
|  | More Likely | Minority Race | 0.088 | 0.766 |
|  |  | Conc. Yes | 1.052 | 0.305 |
|  |  | Hyp. Yes | 0.714 | 0.398 |

Note: “Other” self-identifications for gender were recoded in two ways – combining with ‘male’ and combining with ‘female.’ F statistics and p-values in this table reflect the results of a Wald test comparing coefficients produced under each recode strategy.

Table S4: Weighted Proportions of Regulation Responses

| **Variable** | **Proportion** | | |
| --- | --- | --- | --- |
|  | Less Likely | Neither | More Likely |
| Lost Wages | 0.046 | 0.237 | 0.717 |
| Raise Income Limit | 0.067 | 0.281 | 0.652 |
| Dependent Care | 0.069 | 0.288 | 0.643 |
| Travel & Food | 0.053 | 0.233 | 0.714 |

Note: All proportions are weighted.

Table S5: Weighted Dependent Variable Distributions by Regulatory Provision & Sample Subset

| **Dependent Var.** | **Subset** | **Willingness Rating** | | | | | |
| --- | --- | --- | --- | --- | --- | --- | --- |
|  |  | Less Likely | | Neither | | More Likely | |
| Travel & Food | Full Sample | 0.053 | (0.013) | 0.233 | (0.020) | 0.714 | (0.022) |
|  | White Race/Eth. | 0.015 | (0.006) | 0.243 | (0.023) | 0.741 | (0.023) |
|  | Minority Race/Eth. | 0.089 | (0.025) | 0.223 | (0.033) | 0.688 | (0.038) |
|  | >HS Educ. | 0.057 | (0.016) | 0.203 | (0.023) | 0.740 | (0.026) |
|  | <=HS Educ | 0.044 | (0.024) | 0.300 | (0.040) | 0.656 | (0.042) |
|  | Concrete Yes | 0.032 | (0.018) | 0.259 | (0.037) | 0.709 | (0.038) |
|  | Hypothetical Yes. | 0.026 | (0.014) | 0.161 | (0.028) | 0.813 | (0.031) |
|  | No | 0.103 | (0.032) | 0.284 | (0.040) | 0.613 | (0.044) |
| Raise Income Limit | Full Sample | 0.067 | (0.014) | 0.281 | (0.022) | 0.652 | (0.023) |
|  | White Race/Eth. | 0.025 | (0.008) | 0.322 | (0.025) | 0.654 | (0.026) |
|  | Minority Race/Eth. | 0.108 | (0.026) | 0.242 | (0.034) | 0.650 | (0.039) |
|  | >HS Educ. | 0.078 | (0.018) | 0.253 | (0.025) | 0.668 | (0.028) |
|  | <=HS Educ | 0.041 | (0.019) | 0.344 | (0.042) | 0.615 | (0.043) |
|  | Concrete Yes | 0.065 | (0.025) | 0.263 | (0.036) | 0.672 | (0.039) |
|  | Hypothetical Yes | 0.059 | (0.022) | 0.221 | (0.031) | 0.720 | (0.036) |
|  | No | 0.077 | (0.026) | 0.363 | (0.043) | 0.560 | (0.045) |
| Dependent Care | Full Sample | 0.069 | (0.015) | 0.288 | (0.022) | 0.643 | (0.024) |
|  | White Race/Eth. | 0.018 | (0.007) | 0.328 | (0.025) | 0.654 | (0.026) |
|  | Minority Race/Eth. | 0.118 | (0.028) | 0.250 | (0.035) | 0.632 | (0.039) |
|  | >HS Educ. | 0.068 | (0.018) | 0.274 | (0.026) | 0.658 | (0.028) |
|  | <=HS Educ | 0.073 | (0.028) | 0.319 | (0.040) | 0.608 | (0.043) |
|  | Concrete Yes | 0.073 | (0.026) | 0.327 | (0.041) | 0.600 | (0.042) |
|  | Hypothetical Yes | 0.075 | (0.028) | 0.216 | (0.030) | 0.710 | (0.037) |
|  | No | 0.060 | (0.025) | 0.329 | (0.041) | 0.611 | (0.044) |
| Lost Wages | Full Sample | 0.046 | (0.012) | 0.237 | (0.021) | 0.717 | (0.023) |
|  | White Race/Eth. | 0.019 | (0.007) | 0.236 | (0.023) | 0.745 | (0.024) |
|  | Minority Race/Eth. | 0.071 | (0.021) | 0.238 | (0.035) | 0.690 | (0.038) |
|  | >HS Educ. | 0.050 | (0.015) | 0.224 | (0.025) | 0.725 | (0.027) |
|  | <=HS Educ | 0.036 | (0.015) | 0.267 | (0.040) | 0.697 | (0.041) |
|  | Concrete Yes | 0.092 | (0.026) | 0.225 | (0.037) | 0.683 | (0.040) |
|  | Hypothetical Yes | 0.018 | (0.013) | 0.199 | (0.033) | 0.783 | (0.034) |
|  | No | 0.031 | (0.020) | 0.291 | (0.041) | 0.678 | (0.042) |

NOTE: Weighted proportions for each dependent variable subset are shown without parentheses; standard errors are shown with parentheses. These statistics correspond to Figures 1 and 2 in the main text.

Table S6: Cross-Group Dependent Variable Hypothesis Tests

| **Dependent Var.** | **Independent Var.** | **F** | **p** |
| --- | --- | --- | --- |
| Travel & Food | Race/Eth. | 6.927 | 0.001 |
|  | Education | 1.770 | 0.173 |
|  | Prior Willingness | 4.095 | 0.003 |
| Raise Income Limit | Race/Eth. | 7.646 | 0.001 |
|  | Education | 2.261 | 0.105 |
|  | Prior Willingness | 1.940 | 0.103 |
| Dependent Care | Race/Eth. | 10.104 | <0.001 |
|  | Education | 0.410 | 0.652 |
|  | Prior Willingness | 1.218 | 0.301 |
| Lost Wages | Race/Eth. | 3.856 | 0.022 |
|  | Education | 0.591 | 0.554 |
|  | Prior Willingness | 2.657 | 0.034 |

NOTE: These numbers present the detailed significance test results for the results presented in Figures 1 and 2.

1. Qualtrics. 2021. https://www.qualtrics.com/research-services/online-sample/ [↑](#footnote-ref-1)
2. Qualtrics ESOMAR 28. 2019, pg. 4. [↑](#footnote-ref-2)
